# E2F1在常见肿瘤中的最新研究进展

**DOI:** 10.3779/j.issn.1009-3419.2020.101.32

**Published:** 2020-10-20

**Authors:** 诚 沈, 珏 李, 帅 常, 国卫 车

**Affiliations:** 610041 成都，四川大学华西医院胸外科 Department of Thoracic Surgery, West China Hospital, Sichuan University, Chengdu 610041, China

**Keywords:** E2F1, 肿瘤, 细胞周期相关转录因子, E2F1, Tumor, Cell cycle-related transcription factor

## Abstract

细胞周期相关转录因子E2F1（E2F transcription factor 1）是细胞周期相关转录因子E2F家族成员之一，主要参与包括细胞周期进展、DNA修复、DNA复制、细胞分化，增殖和凋亡等多种细胞过程。E2F1在全身多种肿瘤组织和细胞中呈高表达，起着促癌基因的作用，E2F1表达上调与肿瘤的发生、发展、转移及预后密切相关。因此，E2F1有望成为肿瘤治疗的新靶点。本文就E2F1在目前常见肿瘤中的最新研究进展做一综述。

## Abstract (en)

The cell cycle-related transcription factor E2F1 is a member of the cell cycle-related transcription factor E2F family, mainly involved in various cell processes including cell cycle progression, DNA repair, DNA replication, cell differentiation, proliferation, and apoptosis. E2F1 is highly expressed in a variety of tumor tissues and cells, and it plays a role as a cancer-promoting gene. The up-regulation of E2F1 expression is closely related to the occurrence, development, metastasis and prognosis of tumors. Therefore, E2F1 is expected to become a new target for cancer treatment. This article reviews the latest research progress of E2F1 in current common tumors.

细胞周期相关转录因子E2F1（E2F transcription factor 1）是细胞周期相关转录因子E2F家族成员之一。E2F是在研究腺病毒时首先发现的，参与包括细胞周期的多个过程，包括了细胞的进展、DNA修复、DNA复制、细胞分化，增殖和凋亡等。近年来研究发现E2F1在多种常见肿瘤中呈高表达，本文就E2F1在常见肿瘤中的作用及最新研究进展作一综述。

## 1 E2F1概述

### 1.1 E2F1基本结构

近年来，核蛋白基因的研究，尤其是核蛋白基因与恶性肿瘤发生发展的关系，一直是研究的热点。*E2F1*基因属于转录因子类的核蛋白基因，定位于人20号染色体11区2带（20 q11.2），长约11 kb，其编码的E2F1蛋白是细胞周期中的一个重要蛋白。目前已发现6个家族成员，分别为E2F1-E2F6，相应基因定位于不同的染色体，所有E2F蛋白都包含高度保守的DNA结合区，与E2F二聚体伴侣蛋白（DP蛋白）结合的二聚体区及与RB蛋白家族结合的活化区。目前发现的DP蛋白有DP21、DP22两种，与E2F蛋白结合形成异二聚体增强E2F的转录活性，E2F1、E2F2、E2F3蛋白只与RB蛋白家族中的RB1蛋白结合，E2F4、E2F5蛋白只与同属RB蛋白家族的p107、p130结合。

### 1.2 E2F1生物学功能

细胞增殖需要经历4个时期，其中G_1_期/S期是调节细胞增殖的关键点。E2F1能够编码转录调节因子，在哺乳动物细胞周期的G_1_期/S期转变过程中扮演着重要角色。在G_1_早期，E2F1因其特异结合Rb形成复合物从而转录活性被抑制。在G_1_中后期，细胞周期蛋白（cyclin）与相应的细胞周期蛋白依赖性激酶（cyclin dependent kinase, CDKs）结合后可激活CDKs而使Rb磷酸化，E2F1从Rb蛋白上释放出来并活化，促使细胞从G_1_期进入S期。E2F1参与多种生理、病理过程，除了使细胞由G_1_期向S期过渡促进细胞增殖外，又能通过p53和非p53依赖途径诱导细胞凋亡。通过p53依赖途径，E2F1活化p14/p19从而抑制p53降解，p53高表达抑制下游CDK活性，从而中断细胞周期的进行，达到诱导细胞凋亡的目的；通过p53非依赖途径，E2F1则通过增强调节基因*p73*、*CASP-3*、*CASP-7*等的活性诱导细胞凋亡。

## 2 E2F1在常见肿瘤中的研究进展

### 2.1 E2F1与神经胶质瘤

胶质瘤是中枢神经系统最常见的原发性恶性肿瘤，尽管经过手术、放疗和化疗等综合治疗，术后复发率极高，且复发肿瘤病理恶性程度升高多见。

Zhi等^[1]^通过研究发现，上皮细胞转化序列2（epithelial cell transforming sequence 2 oncogene, ECT2）在神经胶质瘤中被上调并促进神经胶质瘤细胞的增殖。初步实验显示ECT2与垂体肿瘤转化基因1（pituitary tumor transforming gene 1, PTTG1）呈正相关。具体的机制则是验证了ECT2通过调控去泛素化酶PSMD14的表达而影响E2F1的稳定性，从而影响PTTG1的表达。多家实验室均观察到利用微小RNA（miRNA）作用于E2F1的表达从而对胶质瘤细胞增殖产生影响。Huang等^[2]^发现miR-342-3p和miR-377靶向E2F1，以抑制其在mRNA和蛋白质水平上的表达，从而抑制神经胶质瘤细胞系增殖，并在G_1_期阻滞细胞周期。miR-320与神经胶质瘤也有类似的研究结果^[3]^。

长链非编码RNA（long non-coding RNA, lncRNA）是近期研究的热点，相关研究^[4]^发现，lncRNA DLX6-AS1通过竞争miR-197-5p来缓解作为神经胶质瘤的新型治疗靶标的E2F1，从而促进神经胶质瘤的癌变。Xia等^[5]^也发现，lncRNA FER1L4的表达水平与神经胶质瘤密切相关，在高级别胶质瘤中lncRNA FER1L4的表达比低级别胶质瘤中的表达上调，并且lncRNA FER1L4的高表达预示了神经胶质瘤的不良预后。在其研究中提示，lncRNA FER1L4表达与E2F1 mRNA表达呈正相关。敲低FER1L4表达后，E2F1表达显著下调，而miR-372的表达显著上调。FER1L4可用作竞争性内源RNA，与miR-371相互作用或结合，上调E2F1，从而促进神经胶质瘤细胞的周期和增殖。

结合近年来研究^[4, 6, 7]^成果，表明E2F1能促进胶质瘤细胞的生长，具体表现在E2F1在胶质瘤中的表达随着肿瘤恶性程度的增高而升高；抑制E2F1表达可以明显抑制胶质瘤细胞增殖及代谢能力，充分说明E2F1在神经胶质瘤中为促癌作用。同时，上述研究成果也表明无论是miRNA还是lncRNA具体位点对E2F1的持续激活是造成胶质瘤细胞恶性增殖的关键原因之一，因而其是一个理想的分子靶点。

### 2.2 E2F1与乳腺癌

乳腺癌是女性常见的恶性肿瘤，发病率逐年升高并呈年轻化的趋势。研究认为，人类肿瘤的发生几乎都涉及到E2F1调节通路的改变，E2F1表达失调导致细胞增殖异常是人类肿瘤形成的一个重要特征^[8, 9]^。

有研究发现正常乳腺组织中E2F1蛋白阳性表达率为0%，不典型增生组织中为11.1%，而在乳腺癌组织中，E2F1阳性表达率为47.3%，提示其阳性表达随着正常乳腺组织-不典型增生组织-乳腺癌的进展呈逐渐升高趋势，说明E2F1参与了乳腺癌的发生与进展。ki-67是一种细胞增生相关的蛋白，与细胞周期密切相关，其可以作为反映肿瘤细胞增殖能力的指标已经广泛用于恶性肿瘤研究中，表达强弱可作为反映肿瘤预后的临床指标。最新研究^[10]^发现，miR-361-3p是一种新型的miRNA，其过表达促进乳腺癌细胞的增殖并抑制其凋亡，miR-361-3p也可直接下调E2F1。因此，该研究提示miR-361-3p在乳腺癌中可通过下调E2F1表达促进乳腺癌增殖并抑制细胞凋亡。

国内学者发现，乳腺癌组织E2F1和ki-67阳性表达与腋窝淋巴结转移、肿瘤大小、组织学分级、肿瘤原发灶-淋巴结-转移（tumor-node-metastasis, TNM）分期有关（*P* < 0.05）。E2F1和ki-67在乳腺癌中的阳性表达呈显著正相关^[11, 12]^。在他们的研究中还提到，ER、PR水平是乳腺癌患者内分泌治疗和判断预后的一个重要生物学指标，E2F1蛋白阳性表达率与ER、PR表达状态有关，E2F1蛋白阳性表达率在ER、PR阳性组显著低于ER、PR阴性组，这一研究结果也充分说明E2F1蛋白低表达可能预示着患者预后相对较好^[11]^。王宏坤等^[13]^发现，ECT2直接受E2Fs信号通路的调控，ECT2和E2F1蛋白在乳腺癌中的表达呈显著正相关性。*ECT2*基因在缺乏E2F介导的细胞中表达明显受到抑制，而存在E2F1的细胞中，在细胞分裂的过程中，E2F1能够提供转录调控，使ECT2在S期与其他一些因子相互调节，从而在G_2_期/M期细胞分裂达到顶峰，导致细胞增殖。

上述研究表明，E2F1与乳腺癌的进展密切相关，E2F1在乳腺癌中是促癌作用，即E2F1表达上调促进乳腺癌细胞生长和致瘤性，敲低E2F1表达则通过阻断细胞周期而阻滞细胞增殖^[14-16]^。此外，E2F1可能是乳腺癌的一种有前途的生物标志物和治疗靶点，如Giro-Perafita等^[17]^研究提出的lncRNA RP11-19E11.1可作为乳腺癌药物治疗的对象，了解E2F1的具体作用机制将为乳腺癌的基因靶向治疗提供新思路。

### 2.3 E2F1与肺癌

目前肺癌已成为世界上发病率最高的肿瘤，是全世界癌症相关死亡的主要原因。近来研究^[18]^发现，*RB*抑癌基因其产物pRB在细胞周期调控方面起着十分重要的作用, 而与其作用密切相关的一个因子就是近年来新发现的转录因子E2F1。有研究^[19]^表明，lncRNA小核仁RNA宿主基因3（small nucleolar RNA host gene 3, SNHG3）在非小细胞肺癌（non-small cell lung cancer, NSCLC）中表达上调，同时lncRNA SNHG3是E2F1的直接转录目标，研究者们通过实验发现E2F1通过转化生长因子-β途径和白介素-6/JAK2/信号转导通路，激活了SNHG3并促进了NSCLC中细胞的增殖和迁移。有关lncRNA在NSCLC中的类似研究还包括Meng等^[20, 21]^学者提出了LINC00461/miR-4478/E2F1反馈环促进了NSCLC细胞的增殖和迁移。lncRNA-HIT与E2F1相互作用也可以调节其靶基因，研究^[21]^发现，lncRNA-HIT至少部分地通过调节E2F1在其靶基因的启动子区域中的占有率来影响NSCLC细胞的增殖。lncRNA-HIT-E2F1复合物可能是NSCLC治疗的潜在靶标。丝氨酸-精氨酸蛋白激酶（serine-arginine protein kinase, SRPK）属于一类细胞周期调节激酶，在NSCLC组织中的SRPK表达上调。SRPK2的过表达促进NSCLC细胞增殖和细胞周期停滞，而敲低SRPK2抑制NSCLC细胞系中的增殖并促进细胞周期停滞。SRPK2通过SC35的磷酸化激活了细胞周期蛋白相关蛋白的E2F1转录，从而促进了NSCLC的循环进程^[22]^。

在小细胞肺癌中，E2F1高表达参与了小细胞肺癌的侵袭和转移。有关研究^[23]^发现，E2F1可以抑制小细胞肺癌细胞中的ZEB2表达，E2F1通过调控ZEB2的表达促进小细胞肺癌的上皮间质转化过程，表明E2F1在小细胞肺癌上皮间质转化过程中发挥重要作用，并参与小细胞肺癌的侵袭转移。白细胞介素增强子结合因子2（interleukin enhancer binding factor 2, ILF2）在一些恶性肿瘤中被上调，但是，关于小细胞肺癌中的ILF2，很多仍然未知。Zhao等^[24]^研究提出，ILF2在体外和体内促进小细胞肺癌肿瘤生长。ILF2增加氧化磷酸化表达，并降低葡萄糖的摄入量和乳酸的产生。对ILF2靶标的全基因组分析确定了其受E2F1调控。作者研究发现抑制E2F1表达可以逆转ILF2诱导的肿瘤生长并增强线粒体功能。

### 2.4 E2F1与胃癌

胃癌的发生发展同样也受到细胞周期不同时相多个调控点的调节，其中G_1_期/S期最为重要，直接决定细胞是否进入细胞增殖周期。E2F1则主要调节细胞周期由G_1_期向S期的过渡，是细胞增殖和发育所必需。

miRNA作为表观遗传学的范畴之一，在胃癌中扮演着致癌和抑癌的双重作用，是肿瘤发生的关键因素，且miRNA的表达失调与胃癌的发生率、早期诊断和预后有关。因此，对miRNA与E2F1的生物学相关性研究可能有助于更好地了解胃癌的发病机制，促进胃癌的分子生物学诊断与靶向治疗。林爱琴等^[25]^发现，E2F1转录因子可以控制多个miRNA簇的表达，其自身也被许多miRNA靶向，miR-449通过影响细胞周期阻滞和凋亡以避免过多的E2F1诱导的细胞增殖，从而在细胞周期进程的调节中发挥重要作用。miR-449能通过自动调节反馈机制调控细胞周期负反馈调控通路CDK-RB-E2F1，在此通路中E2F1是miR-449a、miR-449b的直接靶基因，miR-449a、miR-449b能下调pRB-E2F1的表达，同时靶向沉默CDK6与CDK25A，最终导致癌细胞pRB脱磷酸化和停留在G_1_。miR-34c也与胃癌的发生有相关性，具体体现在其上游的E2F1可以抑制miR-34c促进胃癌细胞增殖，同时还可以增强对紫杉醇联合顺铂的耐药性，沉默E2F1有利于提高紫杉醇联合顺铂对胃癌细胞的疗效^[26]^。miRNA-19b/20a/92a对胃癌干细胞（gastric cancer stem cells, GCSCs）有作用，研究^[27]^发现，miRNA-19b/20a/92a通过在转录后水平上靶向E2F1并激活β-catenin信号转导途径，促进了胃癌干细胞的自我更新。miR-218具有抗肿瘤活性，但其功能尚待阐明。在这项有关胃癌的研究中，作者探索了miR-218（miR-218-5p）在胃癌细胞周期进程中的调控和功能。miR-218可以抑制胃癌细胞的增殖，在G_1_期诱导细胞周期停滞，并在体内抑制肿瘤的生长和转移。同时其可以抑制pRb/E2F1信号通路。miR-218表达是直接由E2F1激活miR-218宿主基因*SLIT2*和*SLIT3*，这也体现了miR-218表达的负反馈调控机制^[28]^。

上述实验表明，胃癌发生发展涉及多个基因，E2F1表达水平为胃癌患者提供了一个显著相关的发生机制关键点及预后标志，而胃癌组织中这些差异表达的mRNA和miRNA反过来又主要与细胞周期进展有关，说明miRNA改变的E2Fs蛋白表达是胃癌发生发展的重要因素。也有研究发现，从E2F1在低级别上皮内瘤变组织低表达，而在胃癌组织高表达，充分说明了在胃癌的发生过程中E2F1的激活起到促进作用^[29, 30]^。然而研究也显示E2F1的表达与胃癌组织分化程度、浸润深度、淋巴结转移、有无远处转移及TNM分期之间差异不显著，提示*E2F1*的突变过表达可能是胃癌发生演进中的早期关键步骤，一旦细胞恶性转化完成, 其表达则无明显变化。在为进一步了解E2F1表达通路在胃癌中的功能和临床意义奠定了基础。

### 2.5 E2F1与卵巢癌

卵巢癌（ovarian carcinoma, OC）是最常见的妇科恶性肿瘤之一，是女性生殖系统癌症死亡率最高的疾病。由于大多数的卵巢癌患者在诊断时已经是晚期并且已经种植转移，因此其整体5年存活率约为40%。因此迫切需要新的靶向诊断和特异性治疗方法来改善卵巢癌的预后^[31]^。已有研究^[32]^显示E2F1通过上调促进肿瘤发生的基因产物Bcl-2来促进卵巢癌的发生和发展。关雪等^[31]^研究发现，在卵巢癌中，RhoC已被鉴定是通过microRNA靶向作用的促进肿瘤侵袭和发展的癌基因。同时他们还发现E2F1通过调控miR-519调控RhoC的表达。通过在细胞中转染E2F1后检测RhoC的表达明显上升，而在此基础上转染MiR-106b-5P后，发现RhoC表达明显降低。相反，抑制E2F1后，RhoC表达明显降低。因此，E2F1和RhoC之间存在明显的正相关，并通过miR-106b-5P发挥作用，对卵巢癌起促进作用。研究^[32]^还发现，E2F1可能是卵巢癌耐药的重要靶点，靶向YES相关蛋白（Yes-associated protein, YAP）-E2F1 DNA损伤反应途径可以改善卵巢癌细胞对化疗药物（WEE1激酶抑制剂AZD1775）的敏感性。

E2F1主要位于肿瘤细胞核内，为局灶或弥漫性分布。从正常卵巢组织、良性卵巢肿瘤到交界性卵巢肿瘤以及卵巢癌的发展过程中，E2F1的表达在逐渐增高，即卵巢癌中E2F1的高表达可以起促癌作用^[33, 34]^。有学者^[35]^大胆提出想法，对卵巢肿瘤常规作E2F1免疫细胞检测，可早期发现恶性肿瘤细胞，为病理组织学诊断提供一种辅助手段，从而对卵巢癌做出早期诊断。

### 2.6 E2F1与其他肿瘤

甲状腺癌是甲状腺恶性肿瘤中最常见的类型，它的发生发展涉及多种因素的参与，其中与E2F1的密切关系是肿瘤发生发展的重要因素^[36, 37]^。Zhang等^[38]^研究提出，lncRNA RGMB-AS1在甲状腺乳头状癌中表达上调，lncRNA RGMB-AS1的转录受转录因子E2F1调控，并在干扰E2F1表达后通过qRT-PCR检测到lncRNA RGMB-AS1表达下调，同时甲状腺乳头状癌细胞的增殖、迁移和侵袭能力降低。食管癌是我国常见的恶性肿瘤之一，研究提示食管癌中E2F1是过表达，并且表达量的增加与临床分期、肿瘤浸润深度、淋巴结转移及病灶大小有相关性，差异有统计学意义。这些可能与E2F1高表达使得大量G_0_期/G_1_期细胞进入S期，加速了细胞周期进程，大量肿瘤细胞增殖，导致肿瘤进展，淋巴结转移以及远处转移的发生^[39-41]^。子宫内膜癌是女性常见的恶性肿瘤，近年来呈逐年上升趋势，但其发病较为隐匿，在确诊时多数已属中晚期。E2F1在子宫癌组织中的阳性表达明显高于正常子宫内膜组织中的表达，提示E2F1与子宫内膜样腺癌的发生或发展有关，在癌组织中E2F1表达与肿瘤分级及分期有关，级别越高，分期越高，E2F1表达强度越高，表明E2F1可能与肿瘤的恶性程度有关，从而反映了子宫内膜样腺癌的生物学行为，提示E2F1在子宫内膜样腺癌中起到了促癌基因的作用^[42]^。同时，有研究^[43]^发现，lncRNA H19能够通过竞争性结合miRNA-29a-3p调节E2F1的表达，而且E2F1能够通过调控下游靶基因*p53*突变体参与子宫癌的发生，因此，H19和E2F1可能存在共同的信号通路，在参与子宫癌的发生与发展过程中起着一定的协同作用。

E2F1有促进细胞增殖和诱导细胞凋亡的双重作用，在大多数种类的癌细胞中起促癌作用，然而在前列腺癌中，有研究^[44]^提出E2F1是抑癌作用。E2F1与p53一样，诱导暴露于遗传毒性治疗中的细胞发生凋亡，从而成为一种抗肿瘤的安全机制，成为保护生物体免受恶性转化并抑制肿瘤的形成的作用。利用*E2F1*基因来控制肿瘤生长并使肿瘤细胞对化疗敏感^[45]^。一项回顾性研究^[46]^也提出，E2F1阳性组的乳腺癌患者相比于E2F1阴性组有较少的肿瘤复发和局部或远处转移的比率。

## 3 展望与思考

总结上文，E2F1从Rb蛋白上释放出来并活化，促使细胞从G_1_期进入S期。同时，E2F1在全身多种肿瘤组织和细胞中呈高表达，起着促癌基因的作用，E2F1表达上调与肿瘤的发生、发展、转移及预后密切相关。但在少部分肿瘤中，E2F1又起着相反的作用，因此，E2F1的双面性有望成为肿瘤治疗的关注点和新靶点^[47]^。

近年来研究发现，E2F1除调控细胞周期外，还参与到肿瘤相关的代谢重编程，在肿瘤的发生、发展中起到重要作用^[48]^。在一个基因芯片研究中，一组参与线粒功能的基因被鉴定为*E2F1*的靶基因，*E2F1*基因敲除的小鼠观察到了明显的代谢紊乱，尤其是线粒体功能损害，提示E2F1可能在细胞周期转换的过程中调控能量代谢^[49]^。Shen等^[18]^最新研究也提出，E2F1可能是参与肺癌相关成纤维细胞代谢重编程的关键分子，也对肺癌得发生发展有重要影响。在肾细胞癌中E2F1也可能是其代谢的主要转录激活因子^[50]^。

目前有关E2F1在肿瘤中的作用机制的研究还处于初级阶段，依然存在许多问题需要去解答，比如，E2F1具有促进凋亡的作用，但在大多数癌中表现为促癌作用，可否更多挖掘其抑癌作用，利用现有分子通路去发现与促凋亡相关的分子机制？进一步明确E2F1在更多器官癌灶中的表达情况；针对E2F1相关的靶点，可以设计并研究肿瘤耐药相关的机制；结合E2F1与肿瘤代谢重编程相关性，可作为一个潜在的治疗靶点，对肿瘤的治疗产生积极的影响。

相信随着研究者们对E2F1的相关研究进一步深入，我们将对E2F1的生物学功能有新的认识，不仅从E2F1与肿瘤自身的发生发展角度来探索，同时也可以从E2F1参与肿瘤微环境及代谢的角度来研究，使得E2F1在肿瘤的精准治疗中取得新的进步。
